# Supplementary material for: C. elegans septins regulate a subset of sensory neuronal cilia via cell-non autonomous mechanisms in supporting glia
Source: bioRxiv. 2025 Oct 16:2025.10.15.682306. Preprint. [Version 3] doi: 10.1101/2025.10.15.682306 (PMC12632803; doi:10.1101/2025.10.15.682306)
Supplement: Supplement 1 — Figure S1. Ciliary and dendritic phenotypes for various ciliated head neurons (amphid channel, URX, AWC) in septin disrupted worms. (A) Representative fluorescence images of the head region of single and double septin gene mutants, dye-filled with DiI. (B) Representative fluorescence images of single and double septin gene mutant amphid cilia using a knock-in IFT-74::GFP reporter. Bar; 2 μm. (C) Analysis of URX and AWC neuron dendrite morphology and length using gcy-32p::gfp and str-2p::gfp reporters. Fluorescence images show the entire neuronal cell structure; the wing-shaped AWC cilia are shown in the smaller panels. Graphs show dendrite length measurements for single and double septin gene mutants. Means denoted by wide horizontal lines. ns; not significant (p>0.05); Mann Whitney test (URX); Kruskal-Wallis test with Dunn’s post-hoc analysis (AWC) vs WT). Scale bars; 5 μm (large panels); 2 μm (small panels). Figure S2. UNC-61::GFP subcellular localisation in the head and tail. (A) Tail images of worms with endogenously tagged UNC-61::GFP (green) showing puncta near the neuronal dendrites stained with DiI (red). (B) Airyscan images of the head region of worms with endogenously tagged UNC-61::GFP and the apical cell junction component DLG-1::mScarlet. Scale bars; 10 μm. Tables S1-S4. Strains (S1), primer sequences (S2), CRISPR guide and repair template sequences for unc-61 and dlg-1 knockin gfp/mScarlet-tagged alleles (S3) and unc-61::gfp transgene construct sequences. [file media-1.pdf]

Figure S1

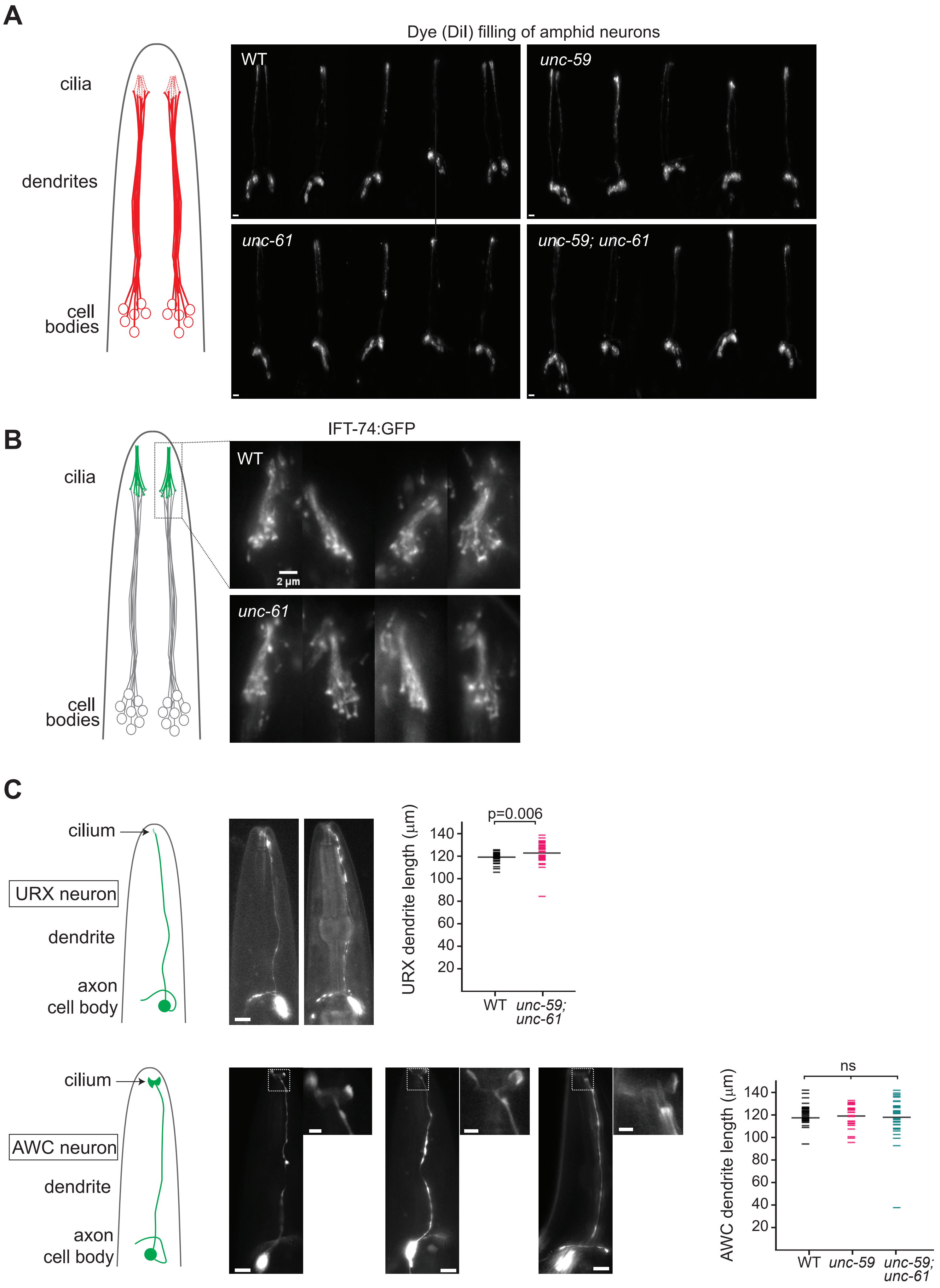
A
Dye (DiI) filling of amphid neurons
cilia
dendrites
cell bodies
WT
unc-59
unc-61
unc-59; unc-61
B
IFT-74:GFP
cilia
cell bodies
WT
2 µm
unc-61
C
cilium
URX neuron
dendrite
axon
cell body
p=0.006
URX dendrite length (µm)
140
120
100
80
60
40
20
WT
unc-59; unc-61
cilium
AWC neuron
dendrite
axon
cell body
ns
AWC dendrite length (µm)
140
120
100
80
60
40
20
WT
unc-59
unc-59; unc-61

Figure S2

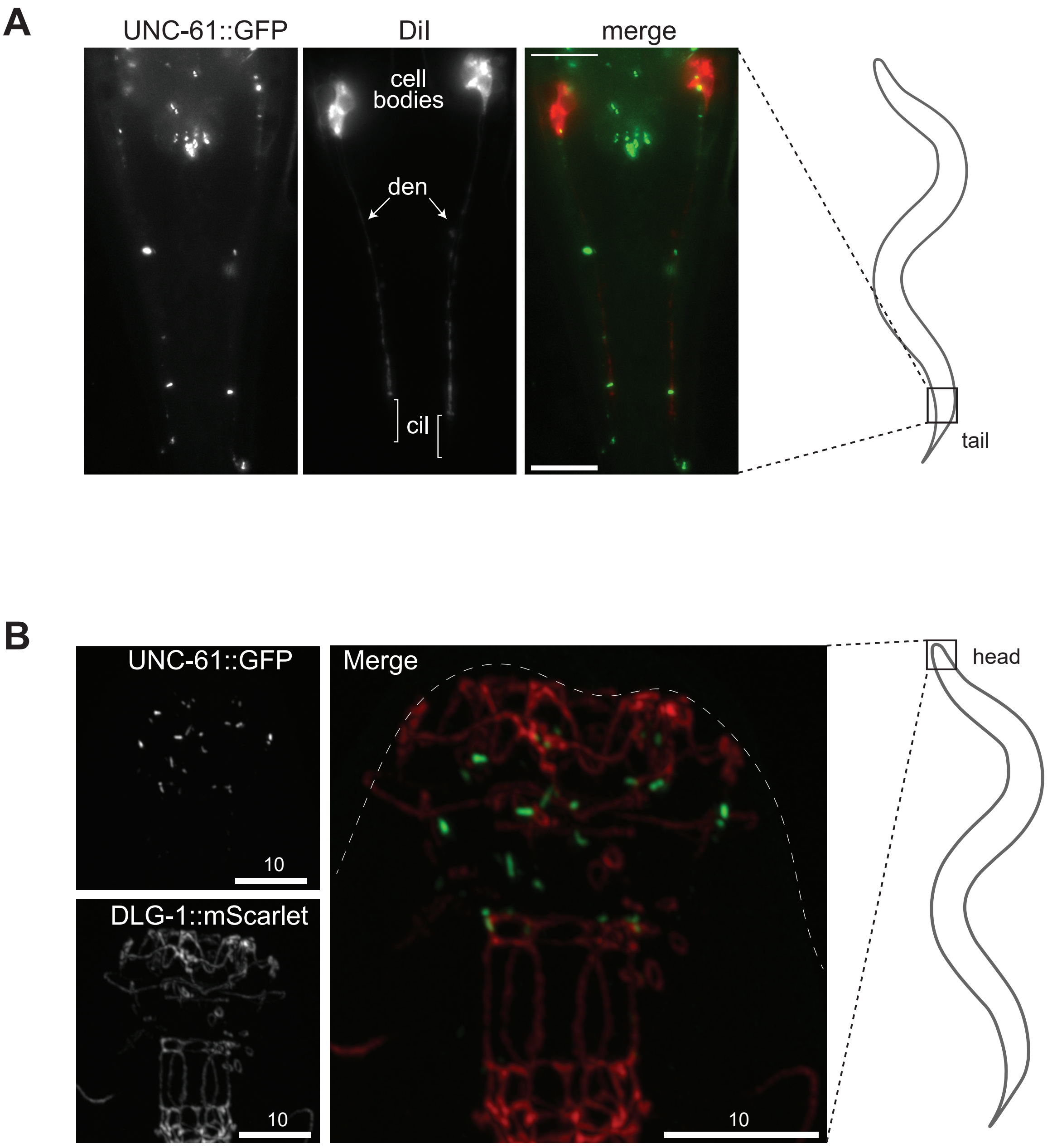
A
UNC-61::GFP
DiI
merge
cell bodies
den
cil
tail
B
UNC-61::GFP
10
DLG-1::mScarlet
10
Merge
10
head

Table S1. Strains used in this study

ID	Genotype	Source
OEB1083	*unc-59(tm1939) I; unc-61(e228) V*	This study
OEB1156	*unc-59(tm1939) I; ift-74(cas499[ift-74::gfp]) II*	This study
OEB1157	*unc-59(tm1939) I; ift-74(cas499[ift-74::gfp]) II; unc-61(e228) V*	This study
OEB1158	*ift-74(cas499[ift-74::gfp]) II; unc-61(e228) V*	This study
OEB1103	*unc-59(tm1939) I; oqIs1 [rpi-2::gfp; xbx-1::tdTomato + pRF4] III*	This study
OEB1119	*unc-59(tm1939) I; oqIs1 [rpi-2::gfp; xbx-1::tdTomato + pRF4] III; unc-61(e228) V*	This study
OEB1104	*oqIs1 [rpi-2::gfp; xbx-1::tdTomato + pRF4] III; unc-61(e228) V*	This study
OEB1099	*unc-59(tm1939) I; mNG::nphp-4 V*	This study
OEB1100	*unc-59(tm1939) I; mNG::jbts-14 X*	This study
OEB1098	*unc-59(tm1939) I; unc-61(e228) V; mNG::nphp-4 V*	This study
OEB1097	*unc-59(tm1939 I; unc-61(e228) V; mNG::jbts-14 X*	This study
OEB1096	*unc-59(tm1939) I; mks-6 (gk674) I; unc-61(e228) V*	This study
OEB1110	*unc-59(tm1939) I; nphp-4(tm925) V*	This study
OEB1111	*unc-59(tm1939) I; mks-6 (gk674) I*	This study
OEB1213	*unc-59(tm1939) I; unc-61(e228) V; nphp-4(tm925) V*	This study
OEB1181	*oqEx951 [mir-228*p*::unc-61::gfp + pRF4]*	This study
OEB1159	*oqEx952 [srb-6*p*::unc-61::gfp + pRF4]*	This study
OEB1186	*unc-59(tm1939) I; unc-61(e228) V; kyIs140 [str-2*p*::gfp + lin-15(+)]*	This study

OEB1117	*unc-59(tm1939) I; kyIs140 [str-2*p::*gfp + lin-15(+)]*	This study
OEB1211	*unc-61(e228) V; oqEx951 [mir-228*p::*gfp::unc::gfp + pRF4]*	This study
OEB1212	*unc-61(e228); oqEx952 [srb-6*p::*unc-61::gfp + pRF4]*	This study
OEB1107	*unc-61(oq173[unc-61::gfp]) V*	This study
OEB1232	*unc-61(oq173[unc-61::gfp]); dlg-1(oq180; [dlg-1::mScarlet]) X*	This study
OEB1341	*ift-74(cas499[ift-74::gfp]) II; unc-61(e228) V;dlg-1(oq180[dlg-1::mScarlet]) X*	This study
OEB1342	*ift-74(cas499[ift-74::gfp]) II; dlg-1(oq180[dlg-1::mScarlet]) X*	This study
OEB1344	*unc-59(tm1939) I; unc-61 (e228) V; iaIs19 [gcy-32*p::*GFP + unc-119(+)]*	This study
FX01939	*unc-59(tm1939) I*	The *C. elegans* Deletion Mutant Consortium et al. (2012)
CB288	*unc-61*(*e228*) V	Brenner (1974)
OEB920	*nphp-4(oq109[mNG::nphp-4) V*	Lange et al. (2021)
OEB938	*jbts-14(oq127[mNG::jbts-14]) X*	Lange et al. (2021)
	nphp-4(tm925) V	
	mks-6(gk674) I	
GOU2362	*ift-74(cas499[ift-74::gfp]) II*	Yi P, et al. 2017
OEB1215	*oqIs1 [pRF4+rpi-2::gfp+xbx-1::tdTomato] III*	formerly *yhEx414*, randomly integrated on Chromosome III
CX3695	*kyIs140 [str-2*p::*gfp + lin-15(+)]*	
ZG611	*iaIs19 [gcy-32*p::*GFP + unc-119(+)]*	

Table S2. Primers used in this study

Primer ID	Name	Sequence	Purpose
**EF029**	unc59.For+144(tm1939)	CTAATGGTTGTTGgtgaggctc	*unc-59 (tm1939)* genotyping
**EF030**	unc59.For-505	cagacccgcgaagtttgaattag	*unc-59 (tm1939)* genotyping
**EF035**	unc59.Rev+687	gactgtcatggtgccaaatg	*unc-59 (tm1939)* genotyping
**EF038**	mks6.For-835	gcgacaaaggtttgatggacag	*mks-6(gk674)* genotyping
**EF039**	mks6.For+1	gcagaaattcagtttccgttg	*mks-6(gk674)* genotyping
**EF040**	mks6.Rev+608	ctacagaatcttacgcgcatgg	*mks-6(gk674)* genotyping
**EF046**	unc61.For+1105ss(e228)	*cgtcgcggaaggtggaata*atgtcaaagc **ttt**	*unc-61 (e228) genotyping (anchor* **allele** foot bridge) see ref. : (Touroutine and Tanis 2020)
**EF047**	unc61.For+1105ss(wt)	*cgtcgcggaaggtggaata*atgtcaaagc **ttc**	*unc-61 (e228) genotyping (anchor* **allele** foot bridge) see ref. : (Touroutine and Tanis 2020)
**EF070**	unc-61.Rev+1972	CGAAGAGTTACAAGATCGAGGGC	*unc-61 (e228)* genotyping
**EF084**	srb-6p.Rev	TTTTATTTCTTCTGTAGAAATTTC	Fusion PCR to generate *srb-6p::unc-61::gfp* construct
**EF085**	srb-6.For-1506	CTTTGCTGCCCGACCCAATGTGG	Fusion PCR to generate *srb-6p::unc-61::gfp* construct
**EF086**	srb-6.For-1469	cgataggcttctggtattgtg	Fusion PCR to generate *srb-6p::unc-61::gfp* construct
**EF091**	unc-61_gfp.For	GAACGTATGAAGTTAATGACTAAG GTCTCCAAGAAGCTCAGAAAGAA GCTTGCATGCCTGCAGGTCGACTC	CRISPR Cas9 of *unc-61::gfp*
**EF092**	unc-61_gfp.Rev	GGGATTAATATCAAAATCATAATAC AGTTCGAATAACAGATTCATTTGTA TAGTTCATCCATGCCATGTGTAATC CC	CRISPR Cas9 of *unc-61::gfp*
**EF093**	unc-61.Rev+2770	GGATTAATATCAAAATCATAATAC	CRISPR Cas9 of *unc-61::gfp*
**EF094**	unc-61.For+2706.P	[Phos]GAACGTATGAAGTTAATGA C	CRISPR Cas9 of *unc-61::gfp*
**EF102**	srb-6p-unc-61.F	GAAATTTCTACAGAAGAAATAAAA ATGAGTTTCGAAACGATTCTC	Fusion PCR to generate *srb-6p::unc-61::gfp* construct

**EF103**	unc-61.Rev+2820	GGGTGTAGTGTGTATTTGAAATAC	Fusion PCR to generate *srb-6*p::*unc-61::gfp* / *mir-228*p::*unc-61::gfp* constructs
**EF105**	miR-228.F-2196	TGCAATGCGGGAAGAGACGA	Fusion PCR to generate *mir-228*p::*unc-61::gfp* construct
**EF106**	miR-228p.R	ATAAGGAGGAAAATGTCTCGCC	Fusion PCR to generate *mir-228*p::*unc-61::gfp* construct
**EF107**	miR-228-unc-61.F	GGCGAGACATTTTCCTCCTTATATGAGTTTCGAAACGATTCTC	Fusion PCR to generate *mir-228*p::*unc-61::gfp* construct
**EF114**	mir-228.F-2171	GATTACTGTATGTGTCGATTTACGG	Fusion PCR to generate *mir-228*p::*unc-61::gfp* construct
**EF139**	dlg-1.mScarletKI.Cterm.F	CCATCATCAGCCGTGAATCGCAGACGCCAATTTGGGTGCCACGTCATTTTGGAACCGGAGGTGGCGGATCTG	CRISPR Cas9 of *dlg-1::mScarlet*
**EF140**	dlg-1.mScarletKI.Cterm.R	GAAGAAACGATTATTTGTCTAAAAAATATCGAGCTTCATCTACTTGTAGAGCTCGTCCATTCCTCCGGTGGAGTGACG	CRISPR Cas9 of *dlg-1::mScarlet*
**EF141**	dlg-1.mScKI.Cterm.R_nested	GAAGAAACGATTATTTGTCTAAAAAAT	CRISPR Cas9 of *dlg-1::mScarlet*
**EF143**	dlg-1.mScarlet.P	[Phos]CCATCATCAGCCGTGAATC	CRISPR Cas9 of *dlg-1::mScarlet*

Table S3. CRISPR Cas9 guides and repair templates

Name	Gene	Location	Sequence
cr-49	*unc-61*	C-term	TCTTAACTTCTTTGACACTT
unc-61::gfp repair template	*unc-61*	C-term	GAACGTATGAAGTTAATGACCAAAGTGTCAAAGAAGTTAAGAAAGAAGCTTGCATGCCTGCAGGTCGACTCTAGAGGATCCCCGGGATTGGCCAAAGGACCCAAAGgtatgtttcgaatgatactaacataacatagaacattttcagGAGGACCCTTGAGGGTACCGGTAGAAAAAATGAGTAAAGGAGAAGAACTTTTCACTGGAGTTGTCCCAATTCTTGTTGAATTAGATGGTGATGTTAATGGGCACAAATTTTCTGTCAGTGGAGAGGGTGAAGGTGATGCAACATACGGAAAACTTACCCTTAAATTTATTTGCACTACTGGAAAACTACCTGTTCCATGGgtaagtttaaacatatatatactaactaaccctgattatttaaattttcagCCAACACTTGTCACTACTTTCTgTTATGGTGTTCAATGCTTcTCgAGATACCCAGATCATATGAAACgGCATGACTTTTTCAAGAGTGCCATGCCCGAAGGTTATGTACAGGAAAGAACTATATTTTTCAAAGATGACGGGAACTACAAGACACgtaagtttaaacagttcggtactaactaaccatacatatttaaattttcagGTGCTGAAGTCAAGTTTGAAGGTGATACCCTTGTTAATAGAATCGAGTTAAAAGGTATTGATTTTAAAGAAGATGGAAACATTCTTGGACACAAATTGGAATACAACTATAACTCACACAATGTATACATCATGGCAGACAAACAAAAGAATGGAATCAAAGTTgtaagtttaaacatgattttactaactaactaatctgatttaaattttcagAACTTCAAAATTAGACACAACATTGAAGATGGAAGCGTTCAACTAGCAGACCATTATCAACAAAATACTCCAATTGGCGATGGCCCTGTCCTTTTACCAGACAACCATTACCTGTCCACACAATCTGCCCTTTCGAAAGATCCCAACGAAAAGAGAGACCACATGGTCCTTCTTGAGTTTGTAACAGCTGCTGGGATTACACATGGCATGGATGAACTATACAAATGAatctgttattcgaactgtattatgattttgatattaatcc
cr-55	*dlg-1*	C-term	GCCACGTCATTAGatgaaat
dlg-1::mScarlet repair template	*dlg-1*	C-term	CCATCATCAGCCGTGAATCGCAGACGCCAATTTGGGTGCCACGTCATtttGGAACCGGAGGTGGCGGATCTGGAGGTGGCGGATCCGTCAGCAAGGGAGAGGCAGTTATCAAGGAGTTCATGCGTTTCAAGGTCCACATGGAGGGATCCATGAACGGACACGAGTTCGAGATCGAGGGAGAGGGAGAGGGACGTCCATACGAGGGAACCCAAACCGCCAAGCTCAAGGTCACCAAGGGAGGACCACTCCCATTCTCCTGGGACATCCTCTCCCCACAATTCATGTACGGATCCCGTGCCTTCACCAAGCACCCAGCCGACATCCCAGACTACTACAAGCAATCCTTCCCAGAGGGATTCAAGTGGGAGCGTGTCATGAACTTCGAGGACGGAGGAGCCGTCACCGTCACCCAAGACACCTCCCTCGAGGACGGAACCCTCATCTACAAGGTCAAGCTCCGTGGAACCAACTTCCCACCAGACGGACCAGTCATGCAAAAGAAGACCATGGGATGGGAGGCCTCCACCGAGCGTCTCTACCCAGAGGACGGAGTCCTCAAGGGAGACATCAAGATGGCCCTCCGTCTCAAGGACGGAGGACGTTACCTCGCCGACTTCAAGACCACCTACAAGGCCAAGAAGCCAGTCCAAATGCCAGGAGCCTACAACGTCGACCGTAAGCTCGACATCACCTCCCACAACGAGGACTACACCGTCGTCGAGCAATACGAGCGTTCCGAGGGACGTCACTCCACCGGAGGAATGGACGAGCTCTACAAGTAGatgaaattggatatttttagacaaataatcgtt

Table S4. Transgenic construct sequences

Construct	Sequence
*srb-6*p*::unc-61::gfp*	cgataggcttctggtattgtgctctttatacttgaaagagcacaacacacctgtgaccagagtcctcatgacaagaatttttttcagtatttgtatatttcgatgagaaaaaaacacactttttgtattttt<br>agtttggtcagatctttgccaaaactgctgaacttttgagcaaaactatttgacgcatgactttcattctttgcgattgatgtgctatgcagattttcttgaaactttagcataccgaaattttattaattaa<br>aagcaattcggtcttttcgaaataattttttaaacctcgattaatatatattacttccttttctcccccttttttatgtatgttttatgattgtgatttccaatacgtgtacattcttgattaatttattctctccag<br>caacaagttgcaaattggaattagagagtcagtgttagagaaatacgagtttccctctttttaatgtaaagtttattgtttcaccactcgtgcaggtgttaacatgctttttcgatcgcccttacgaactttt<br>ttatttatatgtcaataaatattttactgatttcataaaattatttttaatcatttatttactattttaaaaaaaattaaaatgttgcatgattttaataaccaaaattactaattttaaaaagaaacaacact<br>ggttttatgttgtctgatctgagaaattcgttagtgaaatacaagaaaaatagcaaaaataactatgtatgtacaaaataatagggcaccaacaaaaatatttttctgtaatatatttgtcatggttttct<br>ggattttaaaacactcaaaatattatcttgaatgagcaaggcatagcttaatctagagaaaatgatgtgagctgatgtgaacagaaaaaagatacgttccaaaaagtcaccaattttcattgtattatt<br>taccagcattaattttttaaatcaaggtagagtagcgtcagctaggaaattgttaaaacctggataaaattgccagttattataaaaagcatttcaaaaataattttaaattttctaatagtagtcaaa<br>aattggtggttattcagttttgataattcgtaatttaggaaaactaccgtttttttttcaaaattttacaagaagcttttgactagaaattttaaaataactgaaatgttttggagggaatcagtatgaaat<br>ttggtatttttggattattttatatctatttagataaaattggtgccgtttctttgactatttgttgttcaaaagaccagcagaccaaaagctaagcttaaaatttctgcatatatttcagttctttttttacatc<br>gtaaaagaaaaaattctaatctttttacctccattgcgacattttcccagactttatgcatcaaacatacctcctttttttgttttctagaaactactgaaccggatatatgccagtttttattttgtgatcagt<br>cttgaaatttctacagaagaaataaaaATGAGTTTCGAAACGATTCTCTATACTGCATCTGCCCTTCTTTTCATATTTCTATTGGTTCTGCTCACCACCGCTTTTCTCA<br>TCGTCCGACGGTCGAAACAAAGCAGCATAAACCTGCAGACAGTGGTGGTTACCCATGAGAATCCGTATGTCACGATGACCAGTCAATCGTCTAAAATGg<br>tatgaacatctgttgcgcatccattgaaatgacctcgaacatttccaaaaccttgtttaacttctcttttcctcacttacacacatggtttcctccttcttgctaatacatgttttctgaatgcttttctttctct<br>gagtaactcttttatttaaaaaaaaagtctaatttccagGATATCCCAGTGATTCTACCCAAAAGATTCAATATGTCCGACATCGAGCATAAGgtaaattgagaatttttaaacgg<br>tttttcaacaaataattaaagttacagCACTTACCACCTCACCAACCACCTCCACCAGTGCCACATCATCATCAAAACCAGCCAACTCACAATAACACGACTACAAT<br>TTCATCAGCTACGAGCAGCATTAACACCACAACCACGAGCAAGAAGCCAACAATTGCAGCTCCAACGGCTCCTTCACCGATTAAAAGTgtgagtaaattgac<br>tgatttatttggcttaaagctttttagagcgaaccaatcgcattggtaagcccataatcttgccataattatatttttaattatattttctatgttttcagCTCTCAGACCACACTGGCCGTGTTAT<br>GCAACTGAACGGACACGTCGGATTCGATTCTCTTCCTCATCAGCTTGTAAAGAAAGCTGTCGAAGCTGGgtaaccattttcaacattaatttttacttttaaatctaaaa<br>atttcagATTTCAATTCAATCTGATGTGTGTTGGAGAAACGGGAACAGGAAAAACAACACTTATAGAGTCTTTGTTCAACATGAAGCTCGATTTCGAGCCA<br>TGCAATCATGAATTGAAAACTGTTGAGCTGAGAACTTGCACGAAAGgtgagcaacgaaaagtctactgaaacagaaattttgaaattaaatttcagACGTCGCGGAAGG<br>TGGAATACGAGTGAAGCTTCGACTCGTTGAAACTGCCGGATTTGGAGATCAGCTGGATAAAGATAAAAGgtttttatttcagagattttgcgatttttgcgtatccggtc<br>tcgaaacgacaagtttcattgtttttttcaaagtttttcaattaaaaaaaaaagttttatttatttattaaaagctcattcaacaatacacttgaactataacaaattgtgagaaaactacgaaattttataa<br>aaattccgcagcaacgaatatttggaattacagtaatcattgaaagcgcacacttctcgcatttaacaaaaaaattgtcgtgttgagaacgggaaccgtatttttgcagcaaaaatcgcaaaatgatgc<br>gtccgggtgataaaaaaagttatgatctgaaaggaaaaataataattttcagTGCCAAAGTAATTGTCGATTATCTCGAATCGCAGTTTGAAACATACCTCCAAGAAGAGTT<br>GAAGCCACGTCGAATGCTTCAGTATTTCAATGATTCGAGAATCCACGCATGTCTCTACTTCATATCACCTACTGGGCACGGgtaggtggaatatactctgaaatta<br>agtttcagagggttttcagagggttaagctacaaagcttctatttaaaaccgcggtgtgcggcaacaattttgtttttaaaataacatttatttcggctaaaccaattgaaaaatgcacaacatttccaa<br>ctcagagttggaaatgccatagaaatttcctgcaattttcaactttcaggtgatttttgagcatttttcaatcattttaactgaaatcaaaactgttttgtagaattgccgcgctttcctgatttcaaactcta<br>aattgtaactaattttatctaatctttcagACTCAAAGCCCTCGATCTTGTAACTCTTCGCGAATTGGCTAAGCGTGTTAATGTGATCCCAGTGATAGCGAAATCAGA<br>CACAACTTGCAAGGATGAGCTTCTCAGATTCAAAGCGAAAATATTGAGCGAGCTGAAATCTCAGAAAATCGATATTTACACGTTCCCAACTGACGATGA<br>AACTGTCTCAACGACGAACAAGGAAATGAATAAATCGGTTCCGTTCGCCGTTGTTGGAAGTATTGATTTTGTGAAGAAAGAGAATGGACAAATGGTTC<br>GTGCTCGTCAATATCCATGGGGAATAGTCGAAGTGGAGAATGAATCACATTGTGATTTTGTTAAACTCCGTGAAGCACTTCTACGTACAAATGTTGATGA<br>GATGAGACAACGAACTCACGAGTCACTCTATGAAAATTACCGTCGTGACAGACTTCGTCAGATGAAGATTGGAGATGGAGAGACTGGACCAAAGATTA<br>TTGAAAAACTCGCACAGgtattatttcagagaaaatattgtttcgtttcactgattctaagcgtcgattgcagAAACATCGCGAGCATCAAGACGAGTTCAGCCGTCGTGAG<br>CTTACTCTTCGCGAAGAATTTCAGAAGAAGCTTGATGTAACAGAAGGTGACATGAGAAAAGTTGAAGAAGGATTGGCTGCACGCGAGCGAGAGGTTC<br>ATGAGAATTATAATCGAGAGGCGTCGAAACTTGATATGGAGATTCGTCAATTGACTGAAGAACGTATGAAGTTAATGACCAAAGTGTCAAAGAAGTTAA<br>GAAAGAAGCTTGCATGCCTGCAGGTCGACTCTAGAGGATCCCCGGGATTGGCCAAAGGACCCAAAGgtatgtttcgaatgatactaacataacatagaacattttca<br>gGAGGACCCTTGAGGGTACCGGTAGAAAAAATGAGTAAAGGAGAAGAACTTTTCACTGGAGTTGTCCCAATTCTTGTTGAATTAGATGGTGATGTTAAT<br>GGGCACAAATTTTCTGTCAGTGGAGAGGGTGAAGGTGATGCAACATACGGAAAACTTACCCTTAAATTTATTTGCACTACTGGAAAACTACCTGTTCCA<br>TGGgtaagtttaaacatatatatactaactaaccctgattatttaaattttcagCCAACACTTGTCACTACTTTCTgTTATGGTGTTCAATGCTTcTCgAGATACCCAGATCATA<br>TGAAACgGCATGACTTTTTCAAGAGTGCCATGCCCGAAGGTTATGTACAGGAAAGAACTATATTTTTCAAAGATGACGGGAACTACAAGACACgtaagttt<br>aaacagttcggtactaactaaccatacatatttaaattttcagGTGCTGAAGTCAAGTTTGAAGGTGATACCCTTGTTAATAGAATCGAGTTAAAAGGTATTGATTTTAA<br>AGAAGATGGAAACATTCTTGGACACAAATTGGAATACAACTATAACTCACACAATGTATACATCATGGCAGACAAACAAAAGAATGGAATCAAAGTTgta<br>agtttaaacatgattttactaactaactaatctgatttaaattttcagAACTTCAAAATTAGACACAACATTGAAGATGGAAGCGTTCAACTAGCAGACCATTATCAACAA<br>AATACTCCAATTGGCGATGGCCCTGTCCTTTTACCAGACAACCATTACCTGTCCACACAATCTGCCCTTTCGAAAGATCCCAACGAAAAGAGAGACCACA<br>TGGTCCTTCTTGAGTTTGTAACAGCTGCTGGGATTACACATGGCATGGATGAACTATACAAATGAatctgttattcgaactgtattatgattttgatattaatcccattttgtt<br>ttatttccgtttttttatgtatttcaaatacacactacaccc

*mir-228*p::*unc-61*::*gfp*	gattactgtatgtgtcgatttacggcattaattttgagtttgcatgaatccatatatatatatatatatatctcttttgtaaaagtttctgtgttgagtttgcattgtccaatgtttacttaaattagttaaataa<br>tttagttaaataatcctgatgaaactgtaattttactaatcggtgaccctggctgaaaaatgtttattgtgacgtcatactcttgcaaaaacggttgcattgctccgatatggacaacttcccagagcagt<br>gcggtttttgcaagatagcctaaaaagagattcatattcgatttttccctttagacctttccgaagctggccgacaattgtcttgaaaggggtttccaagggataggtggccgagtttttgtattccgcga<br>acacatattaccgttttgggaacttgatagccggcgtcaatttaagaatacgcggaataaaacataaatcagatattatcctacgttaattcaaaatatttttgaaatttgtttaatgtttcagttcaaat<br>ttatgtatctttcgcaaacgtttctgtagttcctttttgctttcgtggaaggattcattttgattgtaaaaaacaaacaaagtttcatcggttagaactccaattaaaatatttttaccatttcccacattata<br>ggaacataagtattcttattttgaagttactttcttggagagtaattaacgggaaaccttatgcgtaaaattttcaaaaattagcatgtttcaattgataagactcaccggataacaaaactatcagtag<br>aattaattaatatgaatgtttttaaaatatgcatgaaacagttttttctcaattcaaaaattattctgacttaatcaagtcataatatttagcataaatattaagtacacctggaacagaagtaacatta<br>atcgtagtttttttgacagaaatgtaccatcgaaaatgattacataaacatttattgtaacatttgagttagttaactggacctaatgaaactgaacattatggcagtgagtctaatgaattttggtctca<br>aactaccaaaagttttcttatattcaggaataccacaaaaaaaacattaaaaactccacatccacgagagttgtattctcaggtagtttcaaattaaactttttctttgatagttttggtcttaaattcatc<br>ggactcactgccattatagacattatttattcggttttttaaagaatttattaaagaactacaatatatgtttcttttattgttggtggaagtttgcgaatttttagactagcagatcctcatctggtaatttt<br>taaaatttacttccacaaactaatttattttgctgttctcacgtatatagaaaataatttaaaaattaaatttgaattcccgcgtaaaaccatttgcgtgcgaaattcaaattcagccactttcgtcgatttt<br>aatggcgaaattttcattggtgacggatgttcccgtttttaaattgttttcatagttgataagctaaggacttaataataagtattttcccttttgaggccttcttaattatgttctgaaacagtagaaata<br>agagattccaccataaatcgtgttcaattcggataatccttgtgtgttcattttcatgacccttcatagtgagggaataattggtctctctctctctctctctctctctctctctctctctcttttttct<br>ctaaaactctctcaatcccttacttttcaacgacctcgacagtatttcaaatgtacacagaacaatagaaatcgcctcgtttctgtttctctctctctctctctctctctctctctctcttacttcttccaa<br>ttgaatcgacaattttcgaaatcgaaataggccacagtgaggaaggccgacgaattgagggaaatacacagaaaccccccgccccataatctgtagagaaaaaagagagaaatgggcggagctt<br>gcttactcttttggcgcctccttctctttctctaagtctcactttccatctgttctttcttctccacatacagtcattcgttggcctgtgctgcctcgtcgactactactactgccttcaaaaattcttttattcgt<br>cgcctcccaaaaactgtttcgcaacttttctattttttttgttgctttggcgagacattttcctccttatATGAGTTTCGAAACGATTCTCTATACTGCATCTGCCCTTCTTTTCATATTT<br>CTATTGGTTCTGCTCACCACCGCTTTTCTCATCGTCCGACGGTCGAAACAAAGCAGCATAAACCTGCAGACAGTGGTGGTTACCCATGAGAATCCGTATG<br>TCACGATGACCAGTCAATCGTCTAAAATGgtatgaacatctgttgcgcatccattgaaatgacctcgaacatttccaaaaccttgtttaacttctcttttcctcacttacacacatggtttcct<br>ccttcttgctaatacatgttttctgaatgcttttctttctctgagtaactcttttatttaaaaaaaagtctaatttccagGATATCCCAGTGATTCTACCCAAAAGATTCAATATGTCCGAC<br>ATCGAGCATAAGgtaaattgagaatttttaaacggtttttcaacaaataattaaagttacagCACTTACCACCTCACCAACCACCTCCACCAGTGCCACATCATCATCAAAA<br>CCAGCCAACTCACAATAACACGACTACAATTTCATCAGCTACGAGCAGCATTAACACCACAACCACGAGCAAGAAGCCAACAATTGCAGCTCCAACGG<br>CTCCTTCACCGATTAAAAGTgtgagtaaattgactgatttatttggcttaaagctttttagagcgaaccaatcgcattggtaagcccataatcttgccataattatatttttaattatattttctatg<br>ttttcagCTCTCAGACCACACTGGCCGTGTTATGCAACTGAACGGACACGTCGGATTCGATTCTCTTCCTCATCAGCTTGTAAAGAAAGCTGTCGAAGCTG<br>GgtaaccattttcaacattaatttttacttttaaatctaaaaatttcagATTTCAATTCAATCTGATGTGTGTTGGAGAAACGGGAACAGGAAAAACAACACTTATAGAGT<br>CTTTGTTCAACATGAAGCTCGATTTCGAGCCATGCAATCATGAATTGAAAACTGTTGAGCTGAGAACTTGCACGAAAGgtgagcaacgaaaagtctactgaaac<br>agaaattttgaaattaaatttcagACGTCGCGGAAGGTGGAATACGAGTGAAGCTTCGACTCGTTGAAACTGCCGGATTTGGAGATCAGCTGGATAAAGATAA<br>AAGgtttttatttcagagattttgcgatttttgcgtatccggtctcgaaacgacaagtttcattgtttttttcaaagtttttcaattaaaaaaaaagttttatttatttattaaaagctcattcaacaatacact<br>tgaactataacaaattgtgagaaaactacgaaattttataaaaattccgcagcaacgaatatttggaattacagtaatcattgaaagcgcacacttctcgcatttaacaaaaaattgtcgtgttgaga<br>acgggaaccgtatttttgcagcaaaaatcgcaaaatgatgcgtccgggtgataaaaaagttatgatctgaaaggaaaaataataattttcagTGCCAAAGTAATTGTCGATTATCTCGAA<br>TCGCAGTTTGAAACATACCTCCAAGAAGAGTTGAAGCCACGTCGAATGCTTCAGTATTTCAATGATTCGAGAATCCACGCATGTCTCTACTTCATATCACC<br>TACTGGGCACGGgtaggtggaatatactctgaaattaagtttcagagggttttcagagggttaagctacaaagcttctatttaaaaccgcggtgtgcggcaacaattttgtttttaaaataacatt<br>tatttcggctaaaccaattgaaaaatgcacaacatttccaactcagagttggaaatgccatagaaatttcctgcaattttcaactttcaggtgatttttgagcatttttcaatcattttaactgaaatcaa<br>aactgttttgtagaattgccgcgctttcctgatttcaaactctaaattgtaactaattttatctaatctttcagACTCAAAGCCCTCGATCTTGTAACTCTTCGCGAATTGGCTAAGCG<br>TGTTAATGTGATCCCAGTGATAGCGAAATCAGACACAACTTGCAAGGATGAGCTTCTCAGATTCAAAGCGAAAATATTGAGCGAGCTGAAATCTCAGAA<br>AATCGATATTTACACGTTCCCAACTGACGATGAAACTGTCTCAACGACGAACAAGGAAATGAATAAATCGGTTCCGTTCGCCGTTGTTGGAAGTATTGAT<br>TTTGTGAAGAAAGAGAATGGACAAATGGTTCGTGCTCGTCAATATCCATGGGGAATAGTCGAAGTGGAGAATGAATCACATTGTGATTTTGTTAAACTC<br>CGTGAAGCACTTCTACGTACAAATGTTGATGAGATGAGACAACGAACTCACGAGTCACTCTATGAAAATTACCGTCGTGACAGACTTCGTCAGATGAAG<br>ATTGGAGATGGAGAGACTGGACCAAAGATTATTGAAAAACTCGCACAGgtattatttcagagaaaatattgtttcgtttcactgattctaagcgtcgattgcagAAACATCGC<br>GAGCATCAAGACGAGTTCAGCCGTCGTGAGCTTACTCTTCGCGAAGAATTTCAGAAGAAGCTTGATGTAACAGAAGGTGACATGAGAAAAGTTGAAG<br>AAGGATTGGCTGCACGCGAGCGAGAGGTTCATGAGAATTATAATCGAGAGGCGTCGAAACTTGATATGGAGATTCGTCAATTGACTGAAGAACGTATG<br>AAGTTAATGACCAAAGTGTCAAAGAAGTTAAGAAAGAAGCTTGCATGCCTGCAGGTCGACTCTAGAGGATCCCCGGGATTGGCCAAAGGACCCAAAG<br>gtatgtttcgaatgatactaacataacatagaacattttcagGAGGACCCTTGAGGGTACCGGTAGAAAAAATGAGTAAAGGAGAAGAACTTTTCACTGGAGTTGTC<br>CCAATTCTTGTTGAATTAGATGGTGATGTTAATGGGCACAAATTTTCTGTCAGTGGAGAGGGTGAAGGTGATGCAACATACGGAAAACTTACCCTTAAA<br>TTTATTTGCACTACTGGAAAACTACCTGTTCCATGGgtaagtttaaacatatatatactaactaaccctgattatttaaattttcagCCAACACTTGTCACTACTTTCTgTTATG<br>GTGTTCAATGCTTcTCgAGATACCCAGATCATATGAAACgGCATGACTTTTTCAAGAGTGCCATGCCCGAAGGTTATGTACAGGAAAGAACTATATTTTTC<br>AAAGATGACGGGAACTACAAGACACgtaagtttaaacagttcggtactaactaaccatacatatttaaattttcagGTGCTGAAGTCAAGTTTGAAGGTGATACCCTTGTT<br>AATAGAATCGAGTTAAAAGGTATTGATTTTAAAGAAGATGGAAACATTCTTGGACACAAATTGGAATACAACTATAACTCACACAATGTATACATCATGGC<br>AGACAAACAAAAGAATGGAATCAAAGTTgtaagtttaaacatgattttactaactaactaatctgatttaaattttcagAACTTCAAAATTAGACACAACATTGAAGATGG<br>AAGCGTTCAACTAGCAGACCATTATCAACAAAATACTCCAATTGGCGATGGCCCTGTCCTTTTACCAGACAACCATTACCTGTCCACACAATCTGCCCTTT<br>CGAAAGATCCCAACGAAAAGAGAGACCACATGGTCCTTCTTGAGTTTGTAACAGCTGCTGGGATTACACATGGCATGGATGAACTATACAAATGAatctg<br>ttattcgaactgtattatgattttgatattaatcccattttgttttatttccgtttttatgtatttcaaatacacactacaccc
